# Computational analysis of two novel deep brain stimulation pulsing patterns on a thalamocortical network model of Parkinson’s disease

**DOI:** 10.3389/fnetp.2025.1674935

**Published:** 2025-10-09

**Authors:** AmirAli Farokhniaee, Siavash Amiri

**Affiliations:** ^1^ Department of Psychology, Neuroscience & Behaviour, McMaster University, Hamilton, ON, Canada; ^2^ Didab PVT. Ltd. Co, Tehran, Iran

**Keywords:** deep brain stimulation, beta power, Morgera’s index of synchronization, thalamocortical network, spiking neural networks, short-term synaptic plasticity, Parkinson’s disease, network physiology

## Abstract

Deep brain stimulation (DBS) at high frequencies has revolutionized efforts to alleviate Parkinson’s disease symptoms for approximately 30 years. Since then, there has been vast investigation into the mechanisms of action of DBS. Recently, synaptic suppression was found to play a pivotal role in the fundamental mechanisms underlying DBS. Based on this understanding, researchers introduced two novel DBS pulsing strategies that use a minimal number of stimuli. In contrast to conventional DBS (cDBS) pulsing, which employs continuous high-frequency pulses (>100 Hz), the two novel methods incorporate changes in pulsing frequency and on/off pulsing periods. In this computational study, we investigated the network effects of these two suggested patterns using an updated version of a biophysically realistic thalamocortical network model of DBS. Both suggested pulsing patterns significantly reduced the exaggerated beta power (∼13 Hz–30 Hz oscillations) in the motor cortex, with careful consideration of the intensity of the stimulating pulses. In addition, they significantly reduced the level of network synchronization. We compared these findings with the effects of 20 and 130 Hz cDBS on our network model and did not observe effects contrary to those of 130 Hz cDBS. The two suggested patterns, which were computationally successful in reproducing known DBS network effects, could potentially increase the battery life of DBS device and reduce the microlesion effect associated with long-term cDBS pulsing. These outcomes, however, require confirmation in further studies.

## 1 Introduction

Parkinson’s disease (PD) affects approximately 1% of the world population over the age of 60, and its prevalence continues to increase alongside increasing life expectancy (Reeve, Simcox, and Turnbull, 2014). It is associated with the loss of dopamine-producing neurons in the brain and is characterized by abnormal neural firing within the cortex and basal ganglia regions of the brain. Deep brain stimulation (DBS) is an established therapy for PD and is used clinically to relieve motor symptoms. Although effective in reducing symptoms, DBS is also associated with side effects, movement disturbances, paresthesia, depression, speech dysfunction (Deuschl et al., 2006), technical limitations, and, critically, a lack of clarity on how it alters the processing of information that drives key neural activities.

To date, the majority of research on the symptoms of PD and their control by DBS has focused on the altered basal ganglia function (Wiecki and Frank, 2010; Caligiore et al., 2016). However, recent studies suggest that the key neural activity changes may be mediated and/or driven by the motor cortex (Lindenbach and Bishop, 2013). There are compelling clinical reasons to consider the importance of the motor cortex in the generation of symptoms and cortical stimulation as a potential therapeutic in PD (Caligiore et al., 2016; Arbuthnott and Garcia-Munoz, 2017), knowing the important role of antidromic activation of the cortex via the hyperdirect pathway and alterations in cortical firing patterns in the therapeutic efficacy of DBS (Anderson et al., 2018).

In parallel, there has been a vast investigation into understanding the DBS mechanisms of action during the last 2 decades. Recently, researchers emphasized the depletion of neurotransmitters due to high-frequency DBS, i.e., the synaptic suppression mechanism, as one of the building blocks of the DBS mechanisms of action (Farokhniaee and McIntyre, 2019). This mechanism causes acute effects (in the order of seconds) based on short-term synaptic plasticity principles, that is, the suppression of synaptic communication due to the depletion of neurotransmitter release in response to the application of high-frequency DBS pulses. Consequently, it suppresses the exaggerated increase in neuronal firing in the postsynaptic cells and neighboring neurons.

This fundamental understanding, combined with theoretical calculations, led to the suggestion of two novel patterns that maximize the suppression of neuronal firing while delivering the minimum possible number of stimuli (Farokhniaee and McIntyre, 2019). These two novel patterns have shown promising effects in single neuron simulations; however, they have not been tested on any neuronal network, i.e., populations of neurons. This is essential in understanding the meso- and macro-scale effects of these stimulation patterns to gain better insights into their practical efficacy.

Hence, in this study, we applied these novel patterns on a recently developed network model of DBS, the thalamocortical microcircuit model (TCM), which has manifested known network effects of DBS and gained the attention of many researchers in the field (Farokhniaee and Lowery, 2019; Farokhniaee and Lowery, 2021). The importance of TCM as the platform for testing DBS strategies is due to numerous reasons: the incorporation of the synaptic suppression mechanism, the production of exaggerated beta power (oscillations in the range of 13 Hz–30 Hz) along with synchronization of the action potentials, and finally, the suppression of the elevated beta power, desynchronization of the network spike patterns, and formation of untouched, excited, and inhibited clusters of neurons within the whole network during high-frequency DBS.

In this paper, we analyzed the effects of the suggested DBS patterns on the thalamocortical network by estimating power spectral densities (PSDs) to investigate the formation and deformation of oscillation patterns and, in addition, evaluating Morgera’s index of synchrony (M), which is useful in measuring the amount of synchronization in the network of spiking neurons. Our results indicate that both novel strategies are successful in delivering similar outcomes as conventional DBS patterns at 130 Hz while applied with careful tuning of their intensities, and further interpretations of the results are provided as a discussion in Section 4.

## 2 Materials and methods

### 2.1 Model description

Certain alterations of synaptic weights within and between the thalamus and cortex in a neural mass model of the thalamo-cortex led to elevated beta power in the rat’s motor cortex (Reis et al., 2019), which is a well-known neurophysiological activity in Parkinsonism. On the other hand, a rat model of PD showed the exaggerated synchronized patterns of spiking neurons in addition to the exaggerated beta power (Li et al., 2012). Inspired by these studies, the spiking neuronal network of the thalamo-cortex in PD-like conditions was introduced (Farokhniaee and Lowery, 2019) and developed as a network model of DBS, known as TCM (Farokhniaee and Lowery, 2021), with a recent improved version that provides higher computational efficiency by utilizing a more advanced algorithm (Farokhniaee and Amiri, 2025). The developers of TCM introduced an important underlying mechanism of action of high-frequency DBS, that is, the synaptic suppression due to short-term synaptic plasticity (Farokhniaee and McIntyre, 2019), thereby establishing TCM as a biophysically realistic network model of DBS. As such, TCM exhibited known network effects of DBS that include elevated beta power, exaggerated synchronized patterns of neuronal spikes, the formation of neuronal clusters such as excited and inhibited ones, and optimized intensity of the DBS-induced electric field to cause the most suppression of the elevated beta power.

TCM contains 540 subthreshold noise-driven spiking neurons that obey Izhikevich neuronal dynamics (Izhikevich, 2003) inherently, connected via Tsodyks–Markram synapses (Tsodyks and Markram, 1997). The excitatory populations in the primary motor cortex were distributed into three layers: supragranular or surface (S, 100 neurons), granular or middle (M, 100 neurons), and infragranular or deep (D, 100 neurons), with a shared population of cortical inhibitory neurons (CI, 100 neurons). The thalamocortical relay nucleus (TCR, 100 neurons) and thalamic reticular nucleus (TRN, 40 neurons) form the excitatory and inhibitory populations of the thalamus, respectively. The distributions of neurons in each substructure of the model, along with complete neuron and synapse parameters, are already presented by Farokhniaee and Lowery (2021).

The network dynamics of the model are described by the following set of equations (Farokhniaee and Amiri, 2025):

$${\dot{v}}_{ij}=0.04v_{ij}^{2}+5v_{ij}-u_{ij}+140+I_{ij}+\sum_{j^{\prime }=1}^{6}\sum_{i^{\prime }=1}^{N_{j}}\omega_{i^{\prime }j^{\prime },ij}{PSC}_{i^{\prime }j^{\prime }}\left(t-\Delta_{j,j^{\prime }}\right)+\sum_{k,t_{k}}\mu_{jk}\delta \left(t-t_{k}\right)+\xi \left(t\right)+I_{dbs}\delta_{j3}{\dot{u}}_{ij}=a_{ij}\left(b_{ij}v_{ij}-u_{ij}\right).$$ (1)

If $v_{ij}\geq v_{p_{ij}}+\zeta \left(t\right)$ , then $v_{ij}⟵c_{ij}$ and $u_{ij}⟵u_{ij}+d_{ij}$ .

For each structure of the TCM model*, j = 1, 2, 3, 4, 5,* and *6* corresponds to the structures S, M, D, CI, TRN, and TCR, respectively. For each neuron *i = 1, 2, …, N*_*j*_ in structure *j* (where *N*_*j*_ is the total number of neurons in layer *j*), *v*_*ij*_ is the membrane voltage, and *u*_*ij*_ represents the membrane recovery variable, where *a*_*ij*_, *b*_*ij*_, *c*_*ij*_, and *d*_*ij*_ are each neuron’s parameters, with random changes that provide non-identical neurons in the network. *I*_*ij*_ is the bias current, with *ξ(t)* and *ζ(t)* as white Gaussian noises. The synaptic connections deliver the PSCs to the neurons using a weighting matrix with *ω*_*ij*_ elements. $\sum_{k,t_{k}}\mu_{jk}\delta \left(t-t_{k}\right)$ presents Poissonian background noise, with inputs occurring at time *t*_*k*_ in each structure. In Equation 1, Δ presents the sum of synaptic and axonal transmission delays (Shepherd, G., & Grillner, 2018) with values presented in the original study.

The PSCs are the solutions *I* in Tsodyks–Markram dynamics given by Equation 2.

$$\dot{u}=-\frac{\;u}{\tau_{f}}+U\left(1-u^{-}\right)\delta \left(t-t_{s}-\Delta \right)\dot{x}=-\frac{1-x}{\tau_{d}}-u^{+}x^{-}\delta \left(t-t_{s}-\Delta \right)\dot{I}=-\frac{\;I}{\tau_{s}}+Au^{+}x^{-}\delta \left(t-t_{s}-\Delta \right),$$ (2)

where *x* represents the fraction of available neurotransmitters after synaptic transmission and *u* is the fraction of available neurotransmitter resources ready to be used. *t*_*s*_ is the spike time, *δ* is the Dirac delta function, *U* is the increment of *u* produced by an incoming spike, $\tau_{f}$ , $\tau_{d}$ , and $\tau_{s}$ are the decay (or recovery) time constant of the variable *u, x*, and *I*, respectively. *A* is the absolute synaptic response.

*I*_*dbs*_ is the DBS-induced intracellular transmembrane current that is added only to 50% of layer D (*j* = 3) neurons using the Kronecker delta function. In the case of conventional DBS (cDBS), it is defined as follows:

$$I_{dbs}\left(t\right)=A\sum_{t^{\prime }=0}^{T}\delta \left(t-t^{\prime }\right),t=0,\frac{1}{f_{dbs}},\frac{2}{f_{dbs}},\ldots ,T,$$ (3)

where *A* is the amplitude of the Dirac delta function, *t′* is the pulse event time that repeats every period of DBS, *f*_*dbs*_ is the DBS stimulation frequency, and *T* is the total time of DBS delivery.

The two novel patterns whose effects on the TCM network were investigated are defined as A-DBS and B-DBS. A-DBS includes an initial burst of pulses at the onset of DBS with 20 pulses at 130 Hz, followed by tonic stimulation at 95 Hz. B-DBS, on the other hand, contains 20 pulses at 130 Hz for the initial burst at the DBS onset, which is followed by burst packs of 4 pulses at 130 Hz that are delivered every 37 ms (Farokhniaee and McIntyre, 2019).

### 2.2 Local field potential and power spectral density

The local field potential (LFP) was estimated following the first-order approximation of the electric field due to the postsynaptic currents of the desired neuronal population. In this work, the cortical LFP was simulated as the sum of all excitatory PSCs (EPSC) in the D layer and all inhibitory PSCs (IPSC) in the CI layer scaled by the conductivity of the surrounding gray matter, *σ* ≈ 0.27 (S m)^−1^, and the distance between the recording electrode and each neuron, *r* = 100 µm (Lindén et al., 2011), as shown in Equation 4:

$$\text{LFP}\left(t\right)=\frac{1}{4\pi \sigma r}\left(\sum_{i=1}^{N_{3}}{PSC}_{i}\left(t\right)+\sum_{i=1}^{N_{4}}{PSC}_{i}\left(t\right)\right).$$ (4)

### 2.3 Network synchronization (Morgera’s index of synchrony)

Morgera’s covariance complexity (Morgera, 1985; Kavasseri and Nagarajan 2006) is a linear measure to evaluate the amount of synchronization and is accurate for neuronal populations when the number of samples (simulation time points, *P*) is much larger than the number of neurons (*N*). Let Γ be the matrix that contains the time series of the membrane voltages (spike trains) in its columns 1 to *N*. The rows will then be the samples (*P* rows). Singular value decomposition of Γ yields N eigenvalues, whose variance corresponds to the *i*th component, $\sigma_{i},$ as in Equation 5

$$\sigma_{i}=\frac{\lambda_{i}^{2}}{\sum_{i=1}^{N}\lambda_{i}^{2}},i=1,2,\ldots ,N.$$ (5)

This provides us with the covariance complexity, *C,* which in information theoretic terms is described as in Equation 6:

$$C=-\frac{1}{\log N}\;\overset{N}{\underset{k=1}{\sum }}\sigma_{k}\;\log\sigma_{k}.$$ (6)

Finally, Morgera’s index of synchrony is defined as M=1−C , which has a value between 0 (indicating full random behavior) and 1 (fully synchronized network).

### 2.4 Simulation

We ran the improved version of the TCM model (Farokhniaee and Amiri, 2025) that ensures embedment of all-to-all random connectivity for 12 s on both Windows and Macintosh machines with success. When simulating the model using a CPU, the parallel algorithm was utilized in updating each time-step through the whole network (multicore and multithread computing). In addition to being solved by CPU power, this model can be run on a graphical processing unit (GPU, available only on a Windows machine), which is useful for simulations that include a higher number of neurons and populations. The simulations that run on a GPU show their strength in run time when the number of neurons is much higher than several hundred. Setting a desirable subpopulation to 0 will lead to the deactivation of that subpopulation from the whole network. We neglected the Poissonian background noise when running the simulations. The white Gaussian noise had a mean of 0 and a standard deviation of 0.5. The threshold noise was set to have a mean of 0 and a standard deviation of 0.1.

## 3 Results

### 3.1 Network raster plots, spike trains, and LFP spectral analysis

**FIGURE 1:**
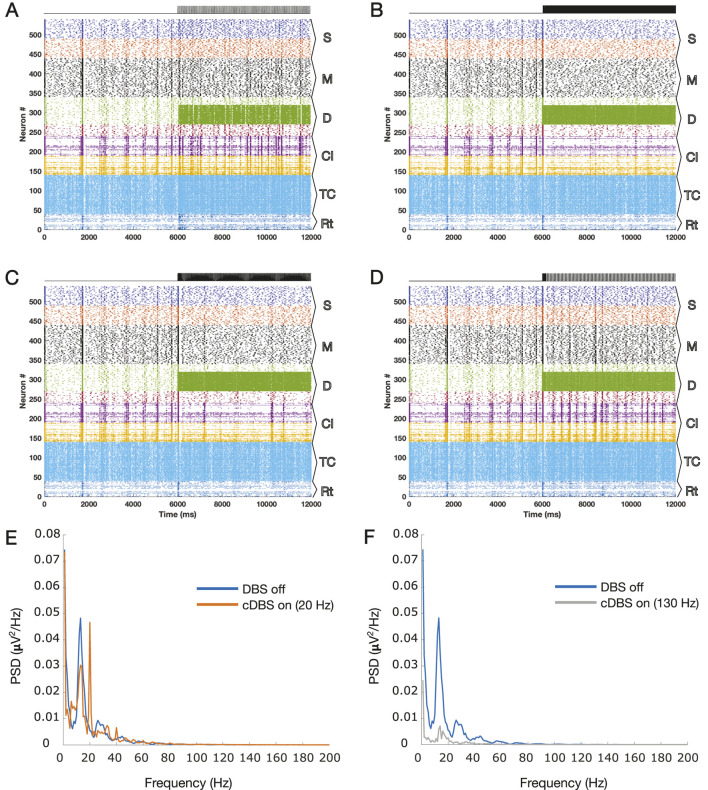
Raster plots of the TCM network for 12 s are presented here with the DBS onset at second 6 for different cDBS frequencies, **(A)** 20 Hz and **(B)** 130 Hz, and the suggested patterns, the **(C)** A-DBS and **(D)** B-DBS protocols. **(E)** PSD corresponding to the LFP estimated for panel A with the harmonics of 20 Hz oscillations shown by arrows, and **(F)** PSD corresponding to the LFP estimated for panel B. The PSDs of panels C and D are shown in Figure 2, where we focused on these two raster plots. The subpopulation layers and nuclei abbreviations are as follows: S, surface or supragranular; M, middle or granular; D, deep or infragranular; CI, cortical inhibitory neurons; TCR, thalamocortical relay; and TRN, thalamic reticular nucleus.

The results of the simulations during different stimulation paradigms (with the onset at second 6) are shown in Figure 1. Figures 1A, B show the results for 20 and 130 Hz cDBS, respectively. A visual comparison of the two diagrams draws attention to the removal of the synchronized events due to 130 Hz cDBS, while they persist during low-frequency DBS at 20 Hz. The PSDs of the simulated LFPs during the off DBS period (the first 6 s of the simulations) on top of the on DBS period (the last 6 s of the simulations) are illustrated in Figure 1E and F for the simulated LFPs of the two panels in Figures 1A, B. The 20 Hz cDBS led to the creation of 20 Hz harmonics (evident as integer multiples of 20 Hz peaks) and apparently an elevated beta power, whereas the 130 Hz cDBS led to the removal of the exaggerated beta power during the off DBS period.

**FIGURE 2:**
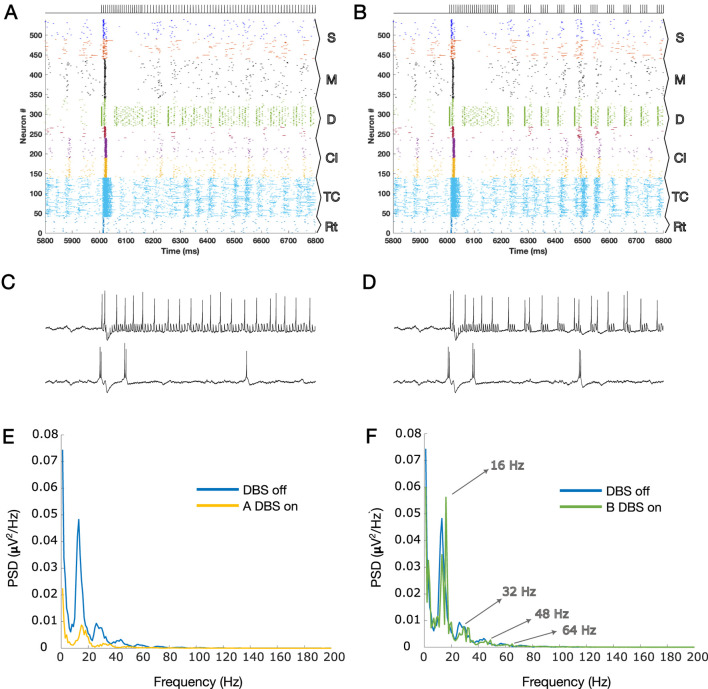
**(A)** Zoomed plot for one second of simulation that contains the onset of the A-DBS pattern (already presented in Figure 1C in a large scale); **(B)** same as A but for B-DBS. **(C, D)** Two spike trains of PY cells in the cortical D layer are shown for panels A and B, respectively. The top spike trains are those that received the stimulation directly, and the bottom ones are those that received it indirectly (via synaptic connections in the network). **(E)** PSDs during the on and off periods of A-DBS, corresponding to the LFP estimated for Figure 1C. **(F)** Same as G but for the B-DBS pattern; Figure 1D. The harmonics of 16 Hz are highlighted by arrows, evident during B-DBS application. The subpopulation layers and nuclei abbreviations are as follows: S, surface or supragranular; M, middle or granular; D, deep or infragranular; CI, cortical inhibitory neurons; TCR, thalamocortical relay; and TRN, thalamic reticular nucleus.

**FIGURE 3:**
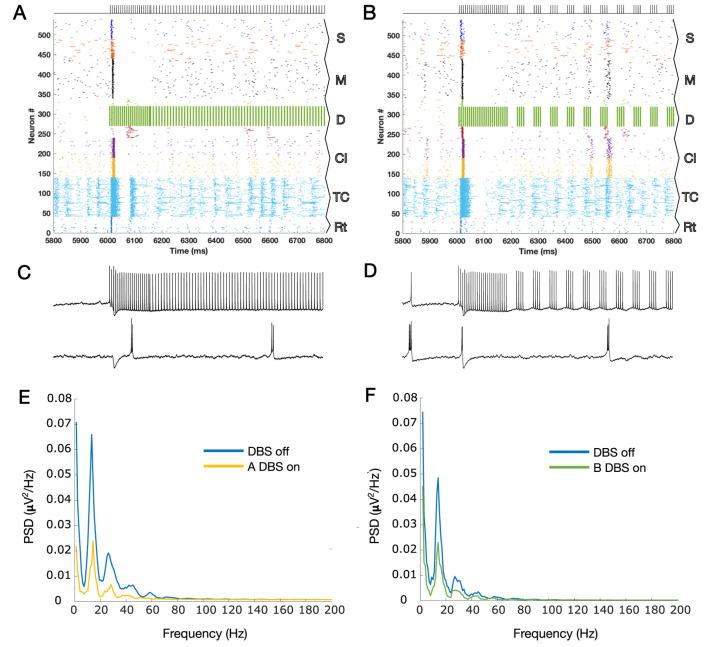
**(A)** Zoomed plot for one second of simulation that contains the onset of the A-DBS pattern, but this time with a higher intensity (×5). **(B)** Same as A but for B-DBS. **(C, D)** Two spike trains of PY cells in the cortical D layer are shown for panels A and B, respectively. The top spike trains are those that received the stimulation directly, and the bottom spike trains are those that received it indirectly (via synaptic connections in the network). **(E)** PSDs during the on and off periods of A-DBS, corresponding to the LFP estimated for panel A, but for the whole 12 s of simulations (6 s off stimulation and 6 s on stimulation). **(F)** Same as G but for the B-DBS pattern, panel B. The harmonics of 16 Hz that were evident in Figure 2H have disappeared. The subpopulation layers and nuclei abbreviations are as follows: S, surface or supragranular; M, middle or granular; D, deep or infragranular; CI, cortical inhibitory neurons; TCR, thalamocortical relay; and TRN, thalamic reticular nucleus.

Figures 1C, D show the results of the application of A-DBS and B-DBS, respectively. For a clearer understanding of these effects, we examined the raster plot panels shown in Figures 1C, D within a 1-s window around the stimulation onset, and the results are shown in Figures 2A, B. We also selected two random pyramidal cell neurons with regular spiking behavior in layer D of the network and illustrated their spike trains in Figure 2C,D for the two different patterns under each corresponding raster plot. In addition, as shown in Figure 1, we investigated the PSD of the two simulated LFPs during off/on periods of A-DBS and B-DBS in Figures 2E, F, respectively. A-DBS appears to have power effects similar to high-frequency 130 Hz cDBS with the attenuation of beta-band activity. However, B-DBS does not appear to attenuate beta power and instead generates harmonics of 16 Hz oscillations (32 Hz, 48 Hz, and so on), which we discuss in Section 4. Combining this information with the spike trains shown in Figure 2C, D, we suggested increasing the amplitude of the stimulation pulses, i.e., *A* shown in Equation 3, by five times in the model to investigate if we would obtain a different effect. Figure 3 shows the results of this change in the amplitude of stimulation with the exact same set-up as shown in Figure 2. In this case, we obtained the attenuation of beta-band activity in both A-DBS and B-DBS patterns (Figures 3E, F).

### 3.2 Beta power attenuation and network synchronization

**FIGURE 4:**
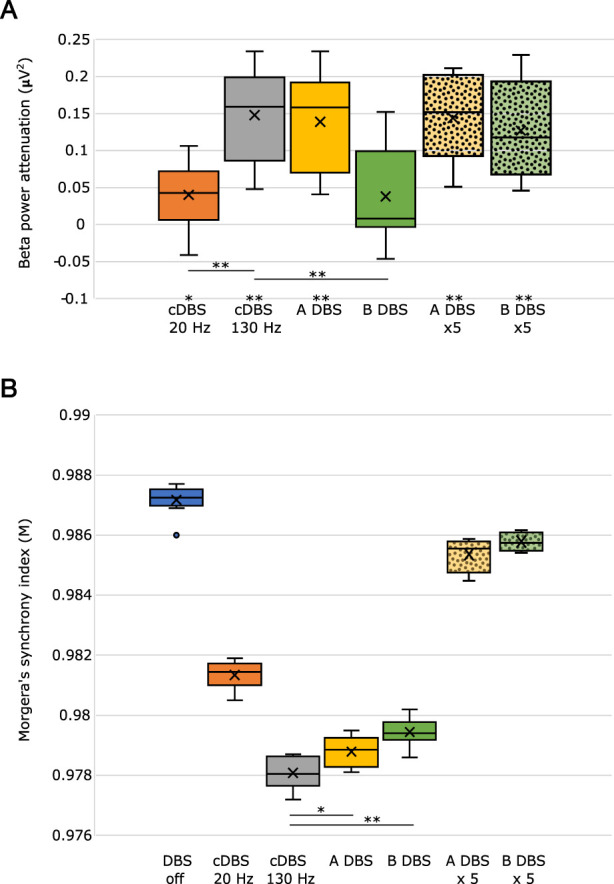
**(A)** Beta power attenuation evaluated during different stimulation protocols. **(B)** Morgera’s index of synchrony estimated during different states, off DBS and on DBS, with different protocols. * indicates *p* < 0.01, and ** indicates *p* < 0.001. The boundaries of the boxplots indicate the 25 and 75 percentiles of the distributions, and the black horizontal lines show their median. The means of the distributions were added as crosses. The dots out of the boxes and their boundaries indicate the outliers. ×5 means five-times higher intensity.

To summarize our results and compare the different stimulation paradigms, we measured the amount of beta power attenuation (which is the difference of beta power due to stimulation from the PD condition (off stimulation)) during the different paradigms with 100 times repetition and showed the results in Figure 4A as boxplots. The statistically significant changes from the PD condition are marked by * and ** for *p* < 0.05 and *p* < 0.01, respectively, utilizing the t-test. A-DBS was shown to provide a similar amount of beta power attenuation as in the application of 130 Hz cDBS, while B-DBS did not show a significant change in beta power with respect to that during the off-stimulation period. However, increasing the intensity of the B-DBS pulses appeared to have a significant change in beta power attenuation and provided similar changes as shown in both A-DBS and 130 Hz cDBS. Notably, we also tested the effect of a 95 Hz cDBS pulsing in our model to check for the necessity of the presence of the initial burst during A-DBS and found that the beta power attenuation is not as significant as that of A-DBS.

In addition to the beta power attenuation, we estimated the synchronization in the network during different stimulations and PD conditions (off stimulation) by evaluating Morgera’s index of synchronization, *M*. cDBS at 130 Hz causes the highest amount of desynchronization in the network (it has the lowest *M*), and A-DBS with no increased amplitude causes the second average highest amount of desynchronization. We found that an increase in the stimulation amplitude, *A*, does not cause a significant desynchronization in the network with respect to the off-stimulation period, although it appeared to be the effective way to reduce the exaggerated beta power, particularly for B-DBS.

## 4 Discussion

In this study, we investigated the cortical network effects of two newly suggested patterns of open-loop DBS based on synaptic suppression mechanism derived from the characteristics of glutamatergic synapses. These two methods (A-DBS: tonic stimulation at a slightly lower frequency than 130 Hz and B-DBS: bursting packs with pauses in between) had already been theoretically derived and well described (Farokhniaee and McIntyre, 2019), and in this article, we applied them to a biophysically realistic model of the thalamo-cortex (Farokhniaee and Lowery, 2021; Farokhniaee and Amiri, 2025). The results of beta power analysis during A-DBS were not contradictory to those of 130 Hz cDBS and, on average, led to the same level of attenuation. However, the B-DBS pattern also achieved the same results only if its intensity was increased by a factor of five. To be more comprehensive and take into account another neurophysiological feature in addition to beta power attenuation analysis, we utilized Morgera’s covariance complexity as a measure of the simultaneous firing of cortical neurons. We found that both A-DBS and B-DBS reduced the network synchronization to the same extent as 130 Hz cDBS, although with statistically significant differences. The increase in the pulsing intensity by a factor of five did not help in reducing the amount of network synchronization. Overall, these findings suggest that the A-DBS pattern is more promising than B-DBS in achieving effects equivalent to high-frequency cDBS. While B-DBS was partially successful in desynchronizing network activity, it produced significant beta attenuation only with careful fine-tuning. Another interesting point that we observed during the application of B-DBS at low intensity was the production of 16 Hz harmonics in the LFP PSD; Figure 2F. Knowing that low-frequency cDBS (such as the one at 20 Hz, shown in Figure 1E) amplifies the driving frequency due to the resonance phenomenon and produces its harmonics, then the same phenomenon takes place for B-DBS as it includes burst packs that periodically repeat eight times for every 500 m, i.e., 16 times per second (16 Hz), with its following harmonics (see Figures 2B, F).

To draw a more robust conclusion from our findings, several points must be considered. First, another set of calculations based on the mean of the synaptic parameters might improve or change the network effects, considering that the parameters of the original study were obtained from a digital reconstruction and simulation of rat neocortical anatomy (Markram et al., 2015), although it seems unlikely since the theoretical calculations (Farokhniaee and McIntyre, 2019) were based on robust synaptic features, which were provided as a sensitivity analysis in the supplementary materials of the referenced work (Farokhniaee and McIntyre, 2019). Second, the assumption to reduce the synaptic transmission by a 50% duty cycle was the basis of the theoretical calculations behind A- and B-DBS patterns, which could vary based on the desired level. Nevertheless, to gain more insights in addition to those found in this study, future works may try investigating the neurophysiological outcomes of the novel patterns in animal experiments such as a rat model, which would help us understand the translational feasibility of these two patterns in practice that, in theory, turned out not to be contradictory with cDBS outcomes. Nevertheless, it is crucial to investigate the neurophysiological outcome of the novel patterns represented in this study to better validate them with real data, such as cortical LFP recordings and electrocorticography (ECoG) from the primary motor cortex. Since the wealth of data in the field of DBS is derived from subthalamic nucleus LFP recordings, the addition of basal ganglia structures to the TCM model would help in further validation of our computational analysis. We hypothesize that the two novel patterns have significant effects not only at the neurophysiological level, including reduction of the microlesion effect, but also in the biomedical engineering aspects of DBS, such as extending the battery life of the implantable pulse generator. These hypotheses should be validated through both theoretical and experimental studies. Amplitude, frequency, and the on–off conditions all affect the energy consumption of the stimulator, and the amount of current needed to have effective outcomes should be carefully compared and quantified across different cases. In addition, the safety ranges of the amplitudes suggested here, such as the increase in B-DBS amplitude by a factor of five, should be carefully measured in future animal-model studies.

## Data Availability

The model presented in this this study can be found in the TCM_DBS repository: https://github.com/aafarokh/TCM_DBS.
